# Normal values for aortic diameters in children and adolescents – assessment *in vivo *by contrast-enhanced CMR-angiography

**DOI:** 10.1186/1532-429X-10-56

**Published:** 2008-12-05

**Authors:** Thomas Kaiser, Christian J Kellenberger, Manuela Albisetti, Eva Bergsträsser, Emanuela R Valsangiacomo Buechel

**Affiliations:** 1 Division of Pediatric Cardiology, University Children's Hospital, Zurich, Switzerland; 2 Department of Diagnostic Imaging, University Children's Hospital, Zurich, Switzerland; 3 Division of Pediatrics, University Children's Hospital, Zurich, Switzerland; 4 Division of Pediatric Oncology, University Children's Hospital, Zurich, Switzerland

## Abstract

**Background:**

Contrast-enhanced CMR angiography (CE-CMRA) is being increasingly used for diagnosing aortic arch anomalies, planning interventions and follow-up assessment. We sought to establish normal values for the diameters of the thoracic aorta and reference curves related to body growth in children using CE-CMRA.

**Results:**

CE-CMRA was performed in 53 children without cardiovascular disease. The median age was 9 years (range 2 – 20 years), weight 30 kg (range 12 – 75 kg), height 131 cm (range 81 – 184 cm), body surface area (BSA) 1.05 m^2 ^(range 0.52–1.9 m^2^). Aortic diameters were measured at nine standardized sites on oblique maximum-intensity projection (MIP) images. Regression analysis of diameters in relation to BSA demonstrated linear relationship between the cross-sectional aortic diameters and the square root of BSA (BSA^0.5^). Normative diameters were (0.57 + 19.37*BSA^0.5^) mm for the aortic sinus, (-3.52 + 18.66*BSA^0.5^) mm for the first segment of the aortic arch, (-3.37 + 16.52*BSA^0.5^) mm for the isthmic region and (-1.27 + 9.89*BSA^0.5^) mm for the descending aorta at the level of the diaphragm. Normative curves are presented.

**Conclusion:**

This study provides normative values for aortic diameters in children measured by CE-CMRA. These data may serve for making the diagnosis of pediatric arch anomalies, assessing the need for treatment and planning interventions.

## Background

Knowledge of the normal dimensions of the thoracic aorta in all its segments is essential for correct diagnosis and management of aortic diseases. In children, aortic anomalies include native coarctation, residual findings after surgery or catheter-guided interventions, connective tissue diseases such as Marfan syndrome and dilatation of the aortic root associated with aortic valve anomalies or occurring after surgery for congenital heart disease, such as the arterial switch operation or the Ross procedure [1-4].

Assessment using cardiovascular magnetic resonance (CMR) is low-invasive, does not involve exposure to radiation and can provide anatomical information of the heart and the thoracic vessels as well as information on ventricular function, blood flow and myocardial perfusion. Contrast-enhanced CMR angiography (CE-CMRA) is particularly useful for evaluation of the aortic arch in its entire course as it provides clear visualization of complex vascular anatomy and reliable measurement of the vessel dimensions [4]. The three-dimensional (3D) CE-CMRA data can be reconstructed into two-dimensional (2D) images in any desired imaging plane, displayed as 2D projection images (maximum intensity projections, MIP) and as volume-rendered images allowing a 3D view of complex malformations, which is particularly appreciated by surgeons.

An excellent agreement between vascular measurements performed on CE-CMRA and conventional angiography has been demonstrated [5]. Therefore, CE-CMRA can be considered as an accurate method for planning catheter-guided interventions and is being increasingly used as a preliminary investigation[6]. With the increasing number of catheter-guided interventions being performed in patients with congenital heart disease, normal data about the vascular structures during growth are becoming more and more important. Normative data for the aortic arch based on conventional angiography, echocardiography and autoptic studies have been published [6-11]. However, to our knowledge, there have been no studies done providing normal data for aortic diameters assessed by CE-CMRA.

The aim of this study was to establish normative values for aortic diameters in children and to provide normograms related to body size.

## Methods

### Subjects

Seventy-seven consecutive children with previous history of a malignancy underwent CE-CMRA for assessment of potential port-a-cath related complications. All subjects were required to have normal cardiovascular anatomy, no evidence of cardiovascular disease and normal body size, according to normative data for the Swiss population [12]. Eight children did not fulfill the inclusion criteria of a normal body size inside the 97% percentile range and/or a sufficient image quality. All subjects presenting conditions, which may have had some influence on the cardiovascular system, were excluded from the study as well. Therefore 9 children after previous radiation therapy involving the chest, 6 undergoing chemotherapeutic treatment at time of CE-CMRA and one presenting with anemia were excluded.

The Ethics Committee of our institution approved the study protocol. Written informed consent allowing additional analysis of the image data for this study was obtained from a legal guardian.

### Technique

All CMR examinations were performed on a 1.5-T Signa MR/i Twinspeed scanner (GE Medical Systems, Milwaukee, Wisconsin, USA) with the smallest coil allowing coverage of the neck and the chest (i.e. quadrature head coil, different sized multichannel phased-array surface coils). CE-CMRA was performed using a 3D fast spoiled gradient echo sequence (3D FSPGR) with linear k-space filling, flip angle 30°, bandwith ± 62 kHz, repetition time 3.2 – 3.4 ms, and echo time 0.9 – 1.1 ms. The field of view (260 – 480 mm), slice thickness (2 – 3.2 mm), and number of partitions (26 – 48) were adjusted to the child's size. A matrix of 256 × 160 and zero interpolation in all three axes (ZIP 512, ZIP 4) provided a spatial resolution ranging from 0.5 × 0.5 × 0.8 mm^3 ^to 0.8 × 0.9 × 1.5 mm^3^. A double dose (0.2 mmol/kg bodyweight, maximum dose 20 ml) of Gadolinium-based contrast medium (dimeglumine gadopentate, Magnevist^®^, Bayer AG, Switzerland; or gadodiamide, Omniscan^®^, GE Healthcare AG, Switzerland) was injected intravenously as a bolus over 10 seconds with a power injector (Medrad Spectris, Pittsburgh, USA), and flushed with the same volume of saline solution and the same injection rate. Image acquisition was timed to the first pass of the contrast medium through the aorta by measuring the contrast travel time (T_C_) to the descending aorta with a real-time two-dimensional fast spoiled gradient echo sequence and a test bolus of 1 ml contrast material. The individual start delay of the 3D FSPGR acquisition was calculated as follows: T_D _= T_C _- T_Ac_/2 + 5, where T_D _is the time delay (in seconds) between the start of the contrast material injection and data acquisition, and T_Ac _is the 3D data acquisition time. Because the primary purpose of the CE-CMRA was assessment of the systemic veins, the 3D data acquisition was repeated three times.

Children under sedation with propofol (n = 23) and those not able to hold their breath (n = 3) were imaged during quiet breathing, while older children were imaged during consecutive breathholds of 14 – 26 seconds duration.

### Image reconstruction and measurements

CE-CMRA source data were reconstructed on a commercially available off-line work station (SUN Microsystems Inc., Mountain View, CA, USA). From the acquired sets of images, the one with the highest signal in the aorta was chosen for reconstruction. Maximum intensity projection (MIP) images and cross-sectional reconstructions were obtained by one investigator (TK) using reformatting software (Volume Analysis 2, Voxtool 3.051f, GE Medical Systems, Milwaukee, Wisconsin, USA).

**Figure 1:**
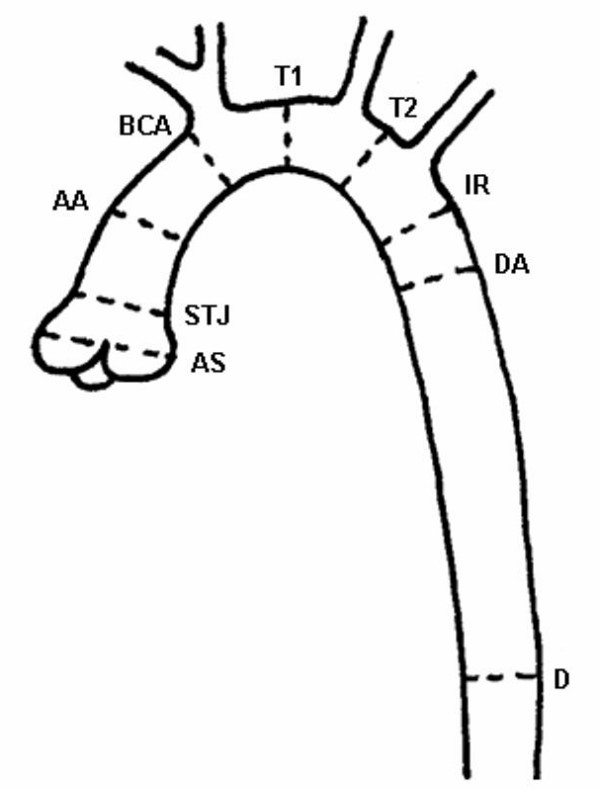
**Sites of measurement**. Aortic sinus (AS), sino-tubular junction (STJ), ascending aorta (AA), proximal to the origin of the brachiocephalic artery (BCA), first transverse segment (T1), second transverse segment (T2), isthmic region (IR), descending aorta (DA), thoracoabdominal aorta at the level of diaphragm (D).

**Figure 2:**
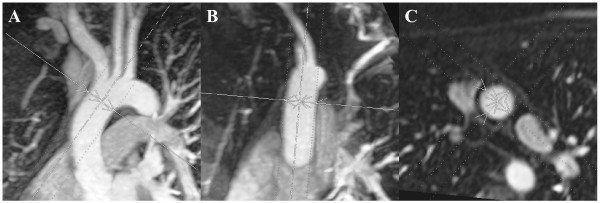
**Imaging planes used for reconstruction of crossectional views**. First MIP, left anterior view (a); second MIP, perpendicular to the first (b); Cross-sectional image, perpendicular to both MIP planes (c).

The aortic diameters were measured at nine standardised sites, consisting of the aortic sinus (AS), sinotubular junction (STJ), ascending aorta at the level of the right pulmonary artery (AA), proximal to the brachiocephalic artery (BCA), first transverse segment (T1), second transverse segment (T2), isthmic region (IR), descending aorta at the level of the left pulmonary artery (DA) and the thoracoabdominal aorta at the level of the diaphragm (D) (figure 1). Each aortic segment was first reconstructed in two double oblique planes. The first plane was set through the longitudinal axis of the vessel corresponding to a left anterior view, as performed in conventional angiograms during catheterization. The second plane was set perpendicularly to the first one along the longitudinal axis of the vessel. Both planes were chosen to be as thick as the vessel itself, in order to assure inclusion of the vessel at its maximal diameter. Finally, a plane perpendicular to both longitudinal views was created and a true cross-section of the vessel obtained with the minimum possible slice thickness (figure 2). Two perpendicular aortic diameters were measured on both the longitudinal images and the cross-sectional images at the corresponding vessel sites.

### Statistical analysis

Demographic data are expressed as median and range.

Measurements from longitudinal MIP images and cross-sectional planes were compared using Bland-Altman analysis [13]. The variability of the measurements was assessed by calculating coefficients of variability (i.e. the standard deviation of the difference divided by their mean). Since aortic cross-sections were observed to be often slightly oval shaped, the shortest diameter passing the centre of the vessel was considered to accurately represent the vessel diameter. The aortic diameters were displayed in relation to BSA^0.5 ^for each measurement site. The best statistical model was defined by a small R2 and analysis of residuals, when comparing the results of regression analysis using linear functions (diameter = a + b*bsa), power functions (diameter = b*bsa^c) (diameter = a + b*bsa^c) and second order polynomial functions (diameter = a+b*bsa + c*bsa^2).

Intra- and interobserver variability were evaluated by using Bland-Altman analysis in 10 randomly selected patients [13]. P-values less than 0.05 were considered statistically significant.

Statistical analysis was performed using a commercially available software package (Prism 4.03, GraphPad Software Inc., San Diego, USA).

## Results

53 children (23 female, 30 male) could be included in the study. The median age was 9 years (range 2–20 years), body weight was 30 kg (range 12–75 kg) and height was 131 cm (range 81–184 cm). The median BSA was 1.05 m^2 ^(range 0.52–1.9 m^2^) calculated using the Mosteller-formula [14]. Age, weight and height did not differ significantly between female and male subgroups.

The range of the diameters for each aortic segment is presented in table 1. A common origin of the brachiocephalic and the left common carotid arteries was observed as a normal anatomical variant in 9 patients (20%). Thus the diameter of the first transversal segment could only be measured in 44 subjects. No additional anatomical variants were observed.

**Table 1:** Range of aortic measurements

	Median	(Range)
AS (mm)	20.7	(13.8 – 31.8)
STJ (mm)	17.5	(11.1 – 26.4)
AA (mm)	18.0	(12.0 – 26.7)
BCA (mm)	17.7	(11.4 – 26.0)
T1 (mm)	16.2	(9.6 – 25.1)
T2 (mm)	14.7	(9.4 – 22.9)
IR (mm)	14.0	(8.8 – 24.9)
DA (mm)	14.4	(9.1 – 22.6)
D (mm)	11.7	(7 – 17.3)

**Figure 3:**
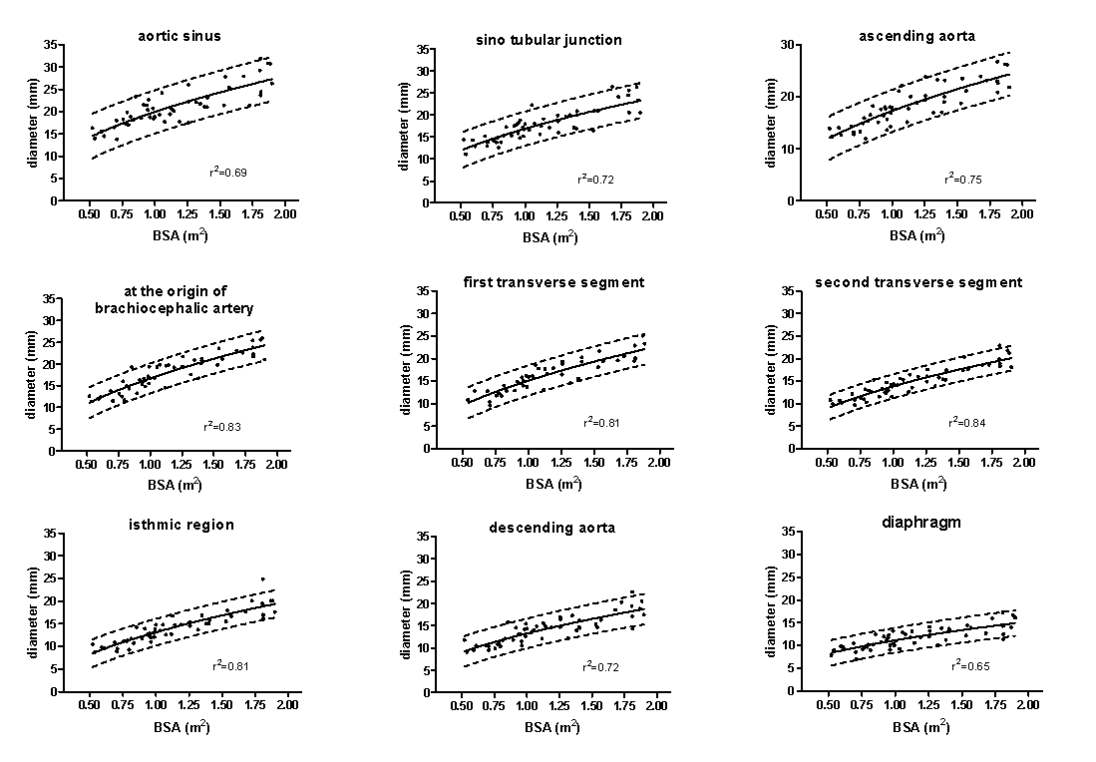
**Reference curves for aortic diameters**. Scatterplots of diameters versus BSA including linear regression graphs with 95% confidence intervals.

A linear relationship between aortic diameters and the square root of BSA was found to be the best model for regression (diameter = a + b*BSA^0.5^). The correlation curves between aortic diameters and BSA for each aortic segment are shown in figure 3. The regression equations and the corresponding standard deviation of residuals are presented in table 2. Z-values for each aortic diameter can be calculated from the values reported in table 2 as follows:

**Table 2:** Functions of normal aortic diameters

**Site**	**Predicted diameter (mm)**	**Standard deviation of residuals (mm)**
**AS**	0.57 + 19.37*BSA^0.5^	2.38
**STJ**	-0.03 + 16.91*BSA^0.5^	1.92
**AA**	-1.33 + 18.6*BSA^0.5^	1.99
**BCA**	-3.38 + 20.07*BSA^0.5^	1.69
**T1**	-3.52 + 18.66*BSA^0.5^	1.63
**T2**	-2.63 + 16.5*BSA^0.5^	1.31
**IR**	-3.37 + 16.52*BSA^0.5^	1.46
**DA**	-1.12 + 14.42*BSA^0.5^	1.64
**D**	1.27 + 9.89*BSA^0.5^	1.34

z-value = (measured diameter – predicted diameter)/(standard deviation of residuals). An electronic calculator of z-scores and graphic display of the measurements for each location is provided with the online version of this paper [see additional file 1].

Bland-Altman comparison of measurements performed on cross-sectional planes and on longitudinal MIPs showed a mean difference of 0.16 mm (1% of mean diameter) and a coefficient of variability of 2.1%.

Inter- and intraobserver variability resulted in mean differences of 1 mm or less for all measurement sites. Coefficients of variation ranged from 3.4% for the isthmic region to 5.5% for the sinotubular junction in one observer, and from 3.4% for the second transverse segment to 6.9% for the sinotubular junction in two observers (table 3).

**Table 3:** Intra- and interobserver variability

	**Intraobserver variability**			**Interobserver variability**		
**Site**	**mean diameter (mm)**	**mean difference (mm)**	**Coefficient of variability (%)**	**mean diameter (mm)**	**mean difference (mm)**	**Coefficient of variability (%)**

**AS**	21.8	0.8	4.6	21.9	0.6	5.6
**STJ**	17.9	0.3	5.5	17.6	1.0	6.9
**AA**	17.7	0.3	4.6	17.7	0.2	5.2
**BCA**	17.6	0.5	3.9	17.5	0.8	4.4
**T1**	16.5	0.6	3.8	16.3	1.0	3.5
**T2**	15.2	0.3	3.7	15.0	0.6	3.4
**IR**	14.9	0.1	3.4	14.8	0.4	4.4
**DA**	14.6	0.2	3.8	14.3	0.8	5.9
**D**	12.4	0.1	4.7	12.1	0.8	6.2

## Discussion

This study provides normative values for the diameters of the thoracic aorta in children and adolescents measured in 9 different segments in vivo using CE-CMRA. These represent the first normal data published for this technique in pediatric patients.

CE-CMRA is increasingly used in clinical practice for assessing thoracic vessels in children and adults with congenital heart disease. Unlike in echocardiography the image quality is independent from any acoustic windows or from the patient's thoracic geometry; the choice of imaging planes is unlimited and three-dimensional images can be provided.

The non-invasiveness and the absence of radiation exposure make this technique ideal for serial follow-up of progressive aortic disease, such as the assessment of aortic dilatation and early detection of aneurysms in patients with Marfan syndrome or other connective tissue diseases and in patients after aortic root surgery such as the arterial switch operation or the Ross procedure. Both the arterial switch operation and the Ross procedure are associated with potential abnormal dilation of the aortic root and ascending aorta [2,15-18]. In children with aortic coarctation, CE-CMRA represents the ideal tool for exact anatomical diagnosis before surgical or catheter-guided treatment, as well as for the assessment of residual lesions during follow up, which include aneurysms and recurrent or residual stenosis [4,5].

Normal data for aortic dimensions have been reported for conventional angiography, echocardiography and post-mortem examinations. The diameters obtained with these methods showed good agreement among the techniques [5,19]. Our results agree well with data from studies using conventional angiography [9-11]. Rammos et al reported diameters slightly smaller for larger children and slightly larger for smaller children than in our study, which may be caused by the different statistical model used (non-linear regression) [11]. Our results correlate well with the data of Clarkson et al, who displayed mean diameters for groups of patients with similar BSA, without performing any regression analysis [10].

For this study we utilized similar measurement sites and statistical model as Sluysmans, who performed echocardiographic measurements of the aorta in a large pediatric population. We found minimally larger diameters for small children, but the values for adolescents were consistent in both studies [7].

Finally, our data correlates very well with autopsy data acquired in small children. A slight difference was observed for data regarding adolescent subjects, and might be caused by the limited number of individuals of this age and body size, that could be studied post-mortem [6].

Body growth is a complex and variable process and its description always represents a simplification. The extensive work done so far for understanding the relationship between somatic growth and development of the cardiac structures suggests that growth of vascular diameters is best described by using a linear relationship between the diameter and the square-root of BSA [7,20]. The different statistical analyses that we performed on our data confirmed this observation; therefore we chose to represent the results graphically as a linear regression between the aortic diameters and BSA^0.5^. Among several formulas than can be used for the calculation of BSA, the one described by Mosteller is known to be accurate for children and is convenient for clinical use [14].

Pulsatility of the vessels is not captured on the static CE-CMRA images. Thus segments close to the aortic root appear less sharply contoured than more peripheral segments, that may be less affected by cardiac motion [21]. This may explain the larger intra- and interobserver variability observed for the segments of the aortic root (i.e. AS and STJ).

By using an adequate slice thickness for the MIP reconstruction in a longitudinal plane at the aortic root, atrial structures may partially superimpose the aorta and make proper visualization of the vascular border difficult. On the basis of these observations, we decided to use cross-sectional measurements for calculation of linear regression in all locations. Measurements of the diameters in both, cross-sectional and longitudinal imaging planes, demonstrated a negligible mean difference of 1% and a limited variability. Moreover, we found most reconstructed cross sections of the aorta to be slightly oval. For clinical use and considering all potential factors which may limit the accuracy of the measurements, including slightly oblique transsection of the vessel when reconstructing, repeatability of the measurements and vessel pulsation, we considered the short diameters to best represent the diameter of the vessel. Analysis of the difference between short diameters and the geometrical means of the short and long diameter resulted in a difference smaller than 1 mm (percentual smaller than 5%), which has to be considered as not significant.

Growth of the thoracic aorta is not limited to childhood and adolescence. Once somatic growth is completed, the thoracic aorta enlarges as a function of age [22]. Mohiaddin et al observed that aortic areas in elderly adults are twice as large as those in teenagers [23]. Data from the Framingham Heart Study show an increment of the diameter at the aortic root of 0.8 mm for women and of 0.9 mm for men for every decade of adult life [22]. Thus, the aortic size in adults seems to be a function of both BSA and age, whereas in childhood aortic growth can be considered to be related solely to body size.

### Limitations

The most important limitation of our study is the lack of data acquired in small children with a BSA smaller than 0.5 m^2^. Sluysmans et al described a linear correlation between the aortic diameters and BSA^0.5 ^for small children with a body size within this range [7]. Although an extrapolation from our data for smaller children would be possible, such extrapolation may potentially increase errors in the calculated regression coefficients. Therefore, we do not recommend the use of our normograms for children smaller than BSA 0.5 m^2^.

We represented both genders in the same regression, since comparison of the normalized diameters for males and females did not show any significant difference in our limited sample size. Differences between the genders may potentially be found in an analysis of a larger cohort of children.

The administration of contrast-medium and the need for sedation in young children raised some ethical scruples for performing this study in absolutely healthy children not requiring an MR examination. By planning the study in children undergoing evaluation of potential port-a-cath complications, we tried to minimize possible factors biasing the normality of our measurements, by applying severe inclusion criteria and excluding all conditions, which may even remotely influence function and growth of the cardiovascular system, such as previous radiation therapy, ongoing chemotherapy or anemia, as described in the methods section. Thus at the time of the study all children recruited were in remission of the disease, did not undergo any treatment or present significant sequels of disease or its treatment.

CE-CMRA is a CMR sequence, which is performed without electrocardiographic gating. The images obtained are static and represent a summation of all cardiac phases. In contrast, measurements at echocardiography or conventional angiography are usually performed during end-systole. This may potentially affect the reliability of a comparison among the techniques. Nevertheless, in a previous study our group could demonstrate high correlation between CE-CMRA and conventional angiographic measurements, even if performed in very small vessels [5].

## Conclusion

This study provides normal values for the aortic diameters in children measured in vivo by CE-CMRA. The results demonstrate that measurements of the aortic diameters in CE-CMRA images are reproducible and correlate well with existing data from other modalities. This data can be used for diagnosing, planning treatment, and follow-up of aortic malformations in children.

## Competing interests

The authors declare that they have no competing interests.

## Authors' contributions

TK carried out image reconstruction and measurements, statistical analysis and preparation of the manuscript. CK performed CE-MRA scans and measurements for assessment of variability. He participated in the design of the study and in the revision of the draft of the manuscript. MA and EB were involved in the design of the study, recruited the patients and collected the clinical data. EVB conceived of and designed the study, was primarily involved in the preparation of the manuscript and its final draft. All authors have read and approved submission of this manuscript. The material in the manuscript has not been published and is not being considered for publication elsewhere in whole or in part in any language.

## Supplementary Material

Additional file 1 Normal aortic shortdiameters. The zip file contains following 3 files for calculation of z-scores and percentiles of aortic measurements. The data provided in the Excel file allows calculation of percentiles and z-scores, as well as graphical display of the measurements on the normative curves. The Word file represents a user's guide giving step by step instructions for installing the certificate to enable the macros included in the previous file xls. The third file contains the certificate for use of the macros included in the Excel file. Click here for file
