# Supplementary material for: Normal values for aortic diameters in children and adolescents – assessment in vivo by contrast-enhanced CMR-angiography
Source: J Cardiovasc Magn Reson. 2008 Dec 5;10(1):56. doi: 10.1186/1532-429X-10-56 (PMC2615773; doi:10.1186/1532-429X-10-56)
Supplement: Additional file 1 — Normal aortic shortdiameters. The zip file contains following 3 files for calculation of z-scores and percentiles of aortic measurements. The data provided in the Excel file allows calculation of percentiles and z-scores, as well as graphical display of the measurements on the normative curves. The Word file represents a user's guide giving step by step instructions for installing the certificate to enable the macros included in the previous file xls. The third file contains the certificate for use of the macros included in the Excel file. [file 1532-429X-10-56-S1.zip › users_guide.doc]

1. Unzip normal_aortic_diameters.zip and save the 3 files contained in it to a folder on your computer.
2. Go to “Start🡺Settings🡺Control Panel”
3. Double click on “Internet Options”
4. Click the “Content” tab then click “Certificates”
5. Click on the “Trusted Root Certification Authorities” tab
6. Click “Import”
7. Browse for ClareJackson.cer (remember which folder you saved the files out of the zip file in).
8. Make sure that you have the following: Place all certificates in the following store “Trusted Root Certification Authorities”
9. You may get a message asking you if you want to add the certificate, click “Yes”
10. You should get a message informing you that your digital certificate was successfully installed and you should be able to see the certificate in the list under the “Trusted Root Certification Authorities” tab
11. Now open normal_aortic_diameters.xls with Excel
